# Green extraction of proteins, umami and other free amino acids from brown macroalgae *Ascophyllum nodosum* and *Fucus vesiculosus*

**DOI:** 10.1007/s10811-021-02569-y

**Published:** 2021-08-24

**Authors:** Viruja Ummat, Marco Garcia-Vaquero, Mahesha M. Poojary, Marianne N. Lund, Colm O’Donnell, Zhihang Zhang, Brijesh K. Tiwari

**Affiliations:** 1 Teagasc Ashtown Food Research Centre, Teagasc, Ashtown, Dublin 15, Ireland; 2 UCD School of Biosystems and Food Engineering, University College Dublin, Belfield, Dublin 4, Ireland; 3 UCD School of Agriculture and Food Science, University College Dublin, Belfield, Dublin 4, Ireland; 4 Department of Food Science, Faculty of Science, University of Copenhagen, Rolighedsvej 26, 1958 Frederiksberg C, Denmark; 5 Department of Biomedical Sciences, Faculty of Health and Medical Sciences, University of Copenhagen, Blegdamsvej 3, 2200 Copenhagen N, Denmark

**Keywords:** Umami, Free amino acids, Algae, Flavour industry, Flavouring agents, Green solvents, Phaeophyta

## Abstract

Seaweeds are a valuable potential source of protein, as well as free amino acids (FAAs) with umami flavour which are in high demand by the food industry. The most commonly used flavouring agents in the food industry are chemically synthesised and therefore are subject to concerns regarding their safety and associated consumer resistance. This study focuses on the effects of extraction time (1 and 2 h) and solvents (0.1 M HCl, 1% citric acid and deionised water) on the extraction of protein and FAAs including umami FAAs from Irish brown seaweeds (*Ascophyllum nodosum* and *Fucus vesiculosus*). Extraction yields were influenced by both the extraction solvent and time, and also varied according to the seaweed used. Both seaweeds investigated were found to be good sources of protein, FAAs including umami FAAs, demonstrating potential application as flavouring agents in the food industry. Overall, the use of green solvents (deionised water and citric acid) resulted in higher recoveries of compounds compared to HCl. The results of this study will facilitate the use of more sustainable solvents in industry for the extraction of proteins and flavouring agents from seaweed.

## Introduction

The world population is projected to reach 9.8 billion in 2050 and 11.2 billion in 2100 (UN 2017) and with these increases the availability of adequate resources to feed the entire population is a major concern. Also, the impact of the current COVID-19 pandemic on global food markets especially those for protein-rich foods is evident (FAO 2020). The general recommendation proposed for protein ingestion by adults is 0.8–1 g kg^−1^ body weight per day, whereas a limit of 0.66 g kg^−1^ day^−1^ has been suggested to avoid protein deficiency (Courtney-Martin et al. 2016). However, with the increased demand for high protein diets, the need to produce protein-rich products has expanded beyond the current nutritional recommendations and has resulted in the food industry increasing the production of protein-enriched foods. For instance, between 2014 and 2017 the proportion of new food and drink product launches claiming high protein composition increased from 1.8 to 4.3% (Fleming 2019).

Over recent years there has also been an increase in the demand for alternatives to meat proteins, due in part to the negative environmental impacts associated with meat production systems, such as greenhouse gas emissions and the requirements for water/land. The production of 1 kg of grain-fed beef requires 5 to 40 times more water than the production of 1 kg of cereal grains, and studies estimate that up to 100 times more water is consumed during the production of meat compared to terrestrial crops (Kumar et al. 2017). Moreover, due to ethical reasons, many people are now transitioning to vegetarianism, veganism and plant-based diets. This has led researchers to focus on different novel sources of proteins, including fungi, algae, by-products from waste processing streams of wheat, legumes and insects amongst others (Pojić et al. 2018), which have the potential to meet consumer needs (Sá et al. 2020). Despite the wide range of alternative sources of protein and amino acids currently being explored, food neophobia may limit the consumption of these new products. Food neophobia is defined as the reluctance of consumers to eat new or unfamiliar foods and is one of the main reasons for failure of new food products launched into the market (Barrena and Sánchez 2013).

Amongst all the new sources of protein, macroalgae or seaweeds represent a relatively untapped market with relatively high consumer acceptance for the production of protein and other value compounds. The protein content of seaweeds varies with the type of algae, for example red species have 20–47% on a dry weight (dw) basis, which is higher than those of green (9–26% dw) and brown (3–15% dw) seaweed species (Fleurence et al. 2018) and also higher than other protein-rich foods such as soybeans, cereals, eggs and fish (Harnedy and FitzGerald 2011). One of the benefits of using seaweed, in spite of it being used as a source for valuable biomolecules, is that the protein yield for the macroalgae is also 2.5–7.5 t ha^−1^ year^−1^, which is 2–5 times higher than the values for wheat or legumes. In addition, macroalgae can be cultivated off-shore and contain all the essential amino acids required for human nutrition (Pangestuti and Kim 2017).

In Southeast Asia, seaweeds are valued for their texture properties and also for their capacity to elicit the sensory perception associated with the umami flavour of food (Mouritsen et al. 2019). The FAAs, or those amino acids that are not bound to other proteins, peptides or amino acids, contribute to the flavour of food (Cherry et al. 2019). These FAAs are generally grouped according to their taste as sweet (threonine (Thr), serine (Ser), glycine (Gly), alanine (Ala) and proline (Pro)), umami (aspartic acid (Asp) and glutamic acid (Glu)), bitter (valine (Val), methionine (Met), isoleucine (Ile), leucine (Leu), phenylalanine (Phe), histidine (His), arginine (Arg) and tryptophan (Trp)) and tasteless (tyrosine (Tyr) and lysine (Lys)) (Poojary et al. 2019). Umami or the fifth basic taste depicts a distinct taste from the other basic tastes (sweet, salty, bitter and sour). It is known that monosodium L-glutamate and L-aspartate elicit umami flavour in food (Lindemann et al. 2002). Increasingly, the food industry has started to focus on producing commercial seaweed products and are investigating several methods to successfully extract seaweed compounds from their complex biological matrices. In spite of their strong industry relevance, only a few studies related to this study have been carried out and limited information on seaweed FAAs is available (Mouritsen et al. 2012, 2019; Saravana et al. 2016; Vieira et al. 2018; Park et al. 2019; Poojary et al. 2019; Milinovic et al. 2020). The extraction of amino acids, including those contributing to umami flavour from seaweeds, has not been fully explored. Most researchers have used dilute HCl to obtain FAAs, which is not preferred for food industry applications. Thus, there is a clear need to develop and validate new clean extraction processes for extraction of these compounds from seaweed that should ideally be fast and efficient, while reducing or eliminating the use of organic solvents following the principles of green chemistry (Anastas and Warner 2000; Chemat et al. 2012).

The aim of this work was to investigate the extraction of protein and FAAs including umami FAAs from the brown macroalgae *Fucus vesiculosus* and *Ascophyllum nodosum* collected in Ireland. Maceration extraction procedures at 80 °C, which are the most widely used currently at industrial large scale, were used to study the effects of different solvents (deionised water, 0.1 M HCl and 1% citric acid (w/v)) and extraction times (1 and 2 h) on the yield of target compounds.

## Materials and methods

### Biological material

*Fucus vesiculosus* and *Ascophyllum nodosum* were harvested in March 2019 in Galway Bay by BEOBIO (Leitir Mór, Connemara, Co. Galway, Ireland). Fresh seaweed samples were cleaned from epitopes and oven-dried (50–60 °C, 2 days) and milled to 1 mm particle size using a hammer mill. The samples were then vacuum-packed and stored under dark conditions at 4 °C for further analysis.

### Chemical reagents

Citric acid monohydrate (≥ 99.8%) and hydrochloric acid (37%) were purchased from Fisher Scientific UK and VWR BDH chemicals, respectively. EDTA standard (9.56% nitrogen) from LECO Corp., USA, was used for LECO analyses. The amino acid calibration standard mix (analytical standard grade) was obtained from Sigma Aldrich (Copenhagen, Denmark). Acetonitrile (HPLC gradient grade) and methanol (HPLC gradient grade) were obtained from VWR International (Søborg, Denmark). Deionised water was used for all the extraction experiments and was obtained from Millipore Milli-Q Direct 8 Water Purification System (Southern Scientific Instruments, USA).

### Extraction conditions

The extraction procedures were performed using 3 solvents: deionised water, 0.1 M HCl and citric acid solution (1%, w/v). These solvents were preheated to 80 °C and then mixed with the milled seaweeds at a ratio of 1:10 (w/v) as described by Kadam et al. (2017). The extraction was carried out using a shaking device (Thermo Scientific MaxQ 6000, USA) at 80 °C and 200 rpm for 1 or 2 h. All extraction procedures were performed in duplicate.

After the extraction, each individual sample was centrifuged at 7000 × *g*, 4 °C and 20 min. The resulting supernatants were stored at − 30 °C until further use.

### Sample analyses

All the analytical procedures were performed in triplicate. The protein content of the extracts was determined using the Dumas method analysing the total nitrogen of the samples in a LECO FP 628 (LECO Corp., USA) following the AOAC method 992.15 (1990). The nitrogen to protein conversion was achieved using 4.17 as a conversion factor as determined by Biancarosa et al. (2017) for brown seaweed. The protein yields were calculated using the formula: Protein yield (%) = [Protein in dried extract (g) × Total weight of dried extract (g)] × [100 /weight of dried seaweed sample (g)]. The % of protein extraction is expressed as g of protein per 100 g of dried seaweed biomass.

The FAA profile of the extracts was analysed following the protocol described by Hildebrand et al. (2020). Briefly, 100 μL of sample was mixed with 12.5% w/v trichloroacetic acid, incubated (4 °C, 1 h) and centrifuged (10,000 × *g*, 20 min) to obtain the supernatant containing FAAs. The samples were neutralised (1 M NaOH), mixed with internal standard (50 μM of 6-aminocaproic acid), filtered through 0.22 μm regenerated cellulose membrane filters and derivatised with o-phthalaldehyde before their injection into a UHPLC-FLD system (Thermo Ultimate 3000 RS, Thermo Scientific, USA) equipped with an Agilent AdvanceBio AAA column (100 mm × 3.0 mm ID × 2.7 μm particle size, Agilent Technologies, USA). The separation was performed using two mobile phases, mobile phase A (10 mM Na_2_HPO_4_ in 10 mM Na_2_B_4_O_7_ decahydrate, pH 8.2) and mobile phase B (mixtures 45:45:10, v:v:v of acetonitrile, methanol and water) at a flow rate of 0.62 mL min^−1^ following a gradient program. The detection was at wavelengths of 340 nm (excitation) and 450 nm (emission). The results are expressed as μg of each amino acid per g dried extract.

### Statistical analyses

The statistical analyses were performed using SPSS version 23.0 (IBM SPSS Statistics). The differences in the recovery of protein, FAAs and umami FAAs by multiple extraction procedures were analysed using general linear models and the differences within groups were analysed using Tukey HSD or Student’s *t*-test where applicable. In all cases, the criterion for statistical significance was *P* ≤ 0.05. The main variance in the data on the recovery of protein and umami FAAs from *A. nodosum* and *F. vesiculosus* was further analysed by principal component analysis (PCA). The PCA was performed using the Varimax rotation method with Kaiser normalisation, weighing and extracting components from the matrix with eigenvalues higher than 1.

## Results and discussion

The extraction conditions used in this study (solvent and extraction time) had a significant influence on the extraction of protein and FAAs including umami FAAs from both seaweed species investigated.

### Effect of extraction parameters on protein yields

**Fig. 1:**
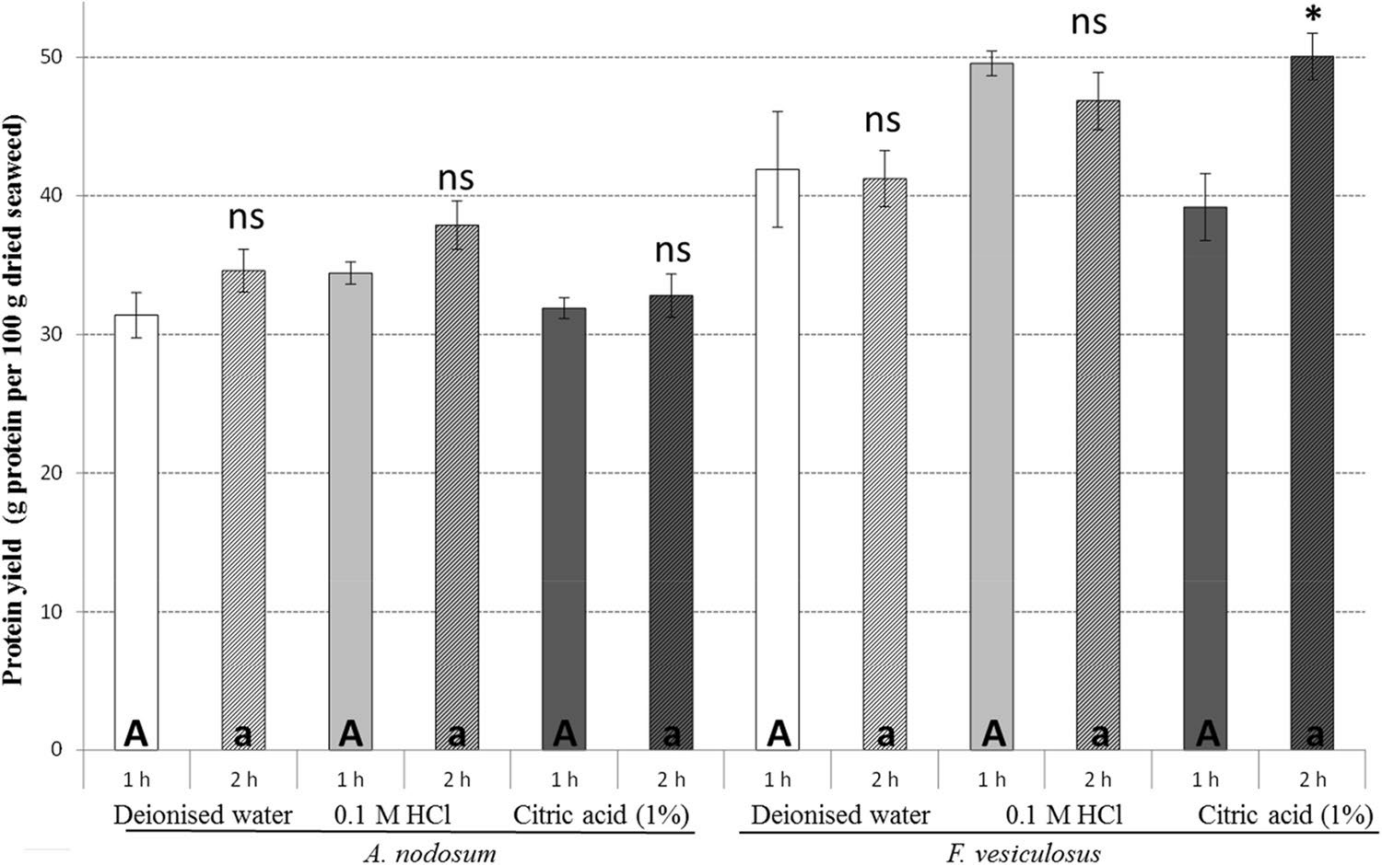
Bar chart representing the effect of solvent type (deionised water, 0.1 M HCl and citric acid (1% w/v)) and extraction time (1 and 2 h) on the yields of protein (g protein per 100 g seaweed biomass or %) extracted from *A. nodosum* and *F. vesiculosus*. Results are expressed as average ± standard deviation of the mean. Different letters indicate statistical differences (*P* < 0.05) between the different extraction treatments using different solvents either 1 h (upper case letters) or 2 h (lower case letters). The statistical differences between 1 and 2 h treatments when using the same solvents are represented on the top of the bars either by ns (non-significant) or * (*P* < 0.05)

Overall, the extraction procedures using different solvents, 0.1 M HCl, 1% (w/v) citric acid and deionised water were effective in obtaining proteins from *A. nodosum* with extraction yields ranging from 31 to 38% (Fig. 1). The maximum protein recoveries for the same extraction time (either 1 or 2 h) were obtained with 0.1 M HCl, followed by deionised water and citric acid. Similarly, previous studies on protein extraction from seaweed using different acid and alkali concentrations reported that overall alkali extraction was better compared to acid extraction. Moreover, focussing only on extraction with HCl, HCl solvents of pH 0.64 extracted more protein from *A. nodosum*, compared to solvents of pH 1.25 (Kadam et al. 2017). However, the differences obtained between the 3 solvents in the current study are not significant; the results are comparable and justify the replacement HCl solvent as it is not food grade. Moreover, in the current study, there was no significant impact of extraction time (1 or 2 h) on the extraction yields of protein. This concurs with the study by Jarpa-Parra et al. (2014) who reported that the extraction time (1 and 2 h) has no significant effect on the protein yields extracted from lentils.

Similar results were observed using *F. vesiculosus*, where maximum protein yield was observed when using 0.1 M HCl as extraction solvent. The extraction time had a significant effect on the recovery of proteins from *F. vesiculosus* when using 1% citric acid, with increased protein recovery observed for an extraction time of 2 h. Similarly to *A. nodosum*, the extraction solvent employed also had a significant effect on the yields of proteins recovered from seaweed. Overall, the recoveries of protein were higher when using citric acid, followed by 0.1 M HCl and water. Overall it was observed that the protein yield obtained from *F. vesiculosus* was higher than that from *A. nodosum*. The main obstacle to efficient protein extraction can be attributed to the ionic interactions between the protein molecules and polysaccharides from the cell wall and intracellular polysaccharides that may differ depending on the seaweed species studied (Harnedy and FitzGerald 2013). Differences in the types and concentration of polysaccharides between *A. nodosum* and *F. vesiculosus* result in differences in the yields and effects of the processing extraction parameters observed between both seaweed species in the current study.

### Effect of the extraction conditions on the extraction of FAAs

The effect of the extraction procedures used on the concentration of FAAs extracted from *A. nodosum* and *F. vesiculosus* is outlined in Tables 1 and 2, respectively. Overall, in the case of *A. nodosum*, the maximum yield of FAAs (5313.91 ± 193.02 μg amino acid g^−1^ dried extract) was obtained using 1% citric acid solvent and for *F. vesiculosus* deionised water resulted in the maximum yield of FAAs (3543.89 ± 7.08 μg amino acid g^−1^). These results demonstrate the feasibility of replacing HCl in extraction processes, by water or other green solvents such as citric acid. There are several factors which influence the solubility of amino acids amongst which the pH is the largest (Tseng et al. 2009). Also differences amongst the amino acids in structure, polarity and charge influence their solubility in water (Tripathy et al. 2018). Therefore, the amounts of amino acids extracted via different solvents can vary.

**Table 1:** Concentration of FAAs (μg amino acid per g dried *A. nodosum* extract) obtained using different solvent types (deionised water, 0.1 M HCl and 1% citric acid) and extraction times (1 and 2 h)

FAAs	Extraction conditions					
	Deionised water		0.1 M HCl		Citric acid (1%)	
	1 h	2 h	1 h	2 h	1 h	2 h
Asp	96.74 ± 2.96	nd	498.51 ± 26.61	1105.66 ± 7.11	481.08 ± 22.68	1534.70 ± 52.57
Glu	447.05 ± 14.68	92.96 ± 2.48	415.97 ± 22.99	2002.87 ± 13.53	134.30 ± 7.42	2038.81 ± 61.31
Ser	nd	nd	32.25 ± 11.33	119.35 ± 1.56	nd	118.37 ± 16.22
His	nd	nd	10.37 ± 1.37	11.64 ± 0.83	9.20 ± 3.33	8.58 ± 1.68
Gly	27.73 ± 1.77	15.09 ± 0.30	68.75 ± 7.24	76.96 ± 0.86	70.95 ± 7.47	112.65 ± 7.74
Thr	nd	nd	nd	58.28 ± 1.44	nd	58.61 ± 2.90
Arg	nd	nd	nd	12.32 ± 1.63	nd	nd
Ala	129.78 ± 3.29	56.53 ± 0.57	288.42 ± 11.05	1257.19 ± 6.03	134.32 ± 5.65	1318.30 ± 42.94
Tyr	nd	nd	10.35 ± 3.00	23.75 ± 5.42	nd	20.27 ± 6.17
Val	nd	6.48 ± 0.30	nd	nd	nd	nd
Met	nd	nd	32.26 ± 5.19	nd	nd	nd
Trp	nd	nd	nd	nd	nd	nd
Phe	2360.00 ± 72.43	nd	2400.69 ± 2.98	123.03 ± 2.98	4224.15 ± 22.37	103.61 ± 5.09
Ile	nd	nd	nd	nd	nd	nd
Leu	6.34 ± 0.50	19.75 ± 0.59	nd	0.99 ± 0.36	nd	nd
Lys	37.90 ± 2.03	6.87 ± 0.45	6.43 ± 3.04	11.62 ± 1.12	nd	nd
Total FAAs	3105.53 ± 55.96 C ***	197.68 ± 2.57 c	3763.99 ± 75.94 B **	4803.66 ± 21.43 b	5055.33 ± 54.45 A	5313.91 ± 193.02 a

Results are expressed as average ± standard deviation of the mean (*n* = 3). The abbreviation nd in the table indicates non-detected. Different letters indicate statistical differences in the amount of total FAAs extracted using multiple solvents during 1 h (uppercase letters) or 2 h (lowercase letters). The statistical differences in the amount of total FAAs extracted by using different times of extraction and the same solvent are represented as: *P < 0.05, **P < 0.01 and ***P < 0.001

**Table 2:** Concentration of FAAs (μg amino acid per g dried *F. vesiculosus* extract) using different solvent types (deionised water, 0.1 M HCl and 1% citric acid) and extraction times (1 and 2 h)

FAAs	Extraction conditions					
	Deionised water		0.1 M HCl		Citric acid (1%)	
	1 h	2 h	1 h	2 h	1 h	2 h
Asp	nd	481.55 ± 7.19	315.96 ± 9.57	314.86 ± 8.0343	3.44 ± 3.06	nd
Glu	112.92 ± 3.81	1469.95 ± 3.86	1142.62 ± 12.21	1157.92 ± 32.08	277.22 ± 57.99	15.86 ± 3.12
Ser	nd	22.42 ± 0.64	23.04 ± 4.88	28.45 ± 2.10	13.93 ± 8.82	nd
His	nd	nd	nd	2.59 ± 1.93	nd	3.19 ± 1.60
Gly	4.94 ± 0.53	62.11 ± 0.34	65.23 ± 2.64	67.08 ± 0.88	36.51 ± 10.41	30.17 ± 0.57
Thr	nd	44.42 ± 1.21	36.30 ± 2.06	38.79 ± 2.68	1.21 ± 1.21	nd
Arg	nd	48.14 ± 0.11	80.85 ± 6.10	91.13 ± 6.01	36.35 ± 9.70	nd
Ala	21.22 ± 0.86	574.75 ± 1.48	532.78 ± 7.37	514.24 ± 6.14	135.93 ± 9.82	142.29 ± 0.97
Tyr	nd	nd	nd	nd	nd	nd
Val	nd	829.95 ± 0.44	1000.35 ± 29.37	1007.01 ± 3.78	5.29 ± 2.39	nd
Met	nd	nd	nd	nd	nd	nd
Trp	nd	nd	nd	nd	nd	nd
Phe	nd	nd	98.19 ± 16.69	63.54 ± 3.89	nd	nd
Ile	nd	nd	nd	nd	nd	nd
Leu	nd	nd	nd	nd	2.38 ± 1.84	nd
Lys	nd	10.55 ± 0.45	23.16 ± 2.08	21.31 ± 1.01	71.30 ± 11.84	62.81 ± 2.11
Total FAAs	139.09 ± 5 C***	3543.89 ± 7.08 a	3318.54 ± 74.66 A	3306.97 ± 50.05 b	583.62 ± 112.92 B*	254.34 ± 4.62 c

Results are expressed as average ± standard deviation of the mean (*n* = 3). The abbreviation nd in the table indicates non-detected. Different letters indicate statistical differences in the amount of total FAAs extracted using multiple solvents during 1 h (uppercase letters) or 2 h (lowercase letters). The statistical differences in the amount of total FAAs extracted by using different times of extraction and the same solvent are represented as: *P < 0.05, **P < 0.01 and ***P < 0.001

The FAA yields obtained also varied with extraction time. In the case of *A. nodosum*, the concentration of FAAs extracted using 0.1 M HCl and 1% citric acid was higher for the 2-h treatments compared to 1-h treatments. In the case of *F. vesiculosus*, higher yields were obtained with 2-h treatment compared to 1-h treatment only for deionised water. Similar to this study, conflicting results on the influence of the extraction time on FAAs from seaweeds have previously been reported. In a study, involving conventional and enzyme-assisted extraction of FAAs from *Saccharina latissima*, *Palmaria palmata* and *Fucus evanescens*, it was observed that the maximum FAAs were extracted at pH 7 when using deionised water, and it was concluded that water is an efficient solvent for the extraction of these compounds (Poojary et al. 2019). Moreover, the authors also noted that in control samples (no enzyme) and β-glucanase-treated *S. latissima*, extraction treatments of up to 1 h resulted in an increased amount of FAAs in the extract, while higher extraction times resulted in a decrease of these compounds (Poojary et al. 2019). However, when using other enzymatic methods for the extraction of FAAs, such as Flavourzyme or Flavourzyme combined with β-glucanase, the yields of extraction of FAAs increased by sixfold following 2 h of incubation with these enzymes (Poojary et al. 2019).

The high lysine concentrations found in the analysed samples is interesting, as seaweeds may be used to balance the amino acid composition of cereal-based products, which often have a low lysine content; being this amino acid currently considered, from a nutritional point of view, as the main limiting amino acid when eating a plant-based protein diet (Sá et al. 2020). As shown in Tables 1 and 2, the maximum contents of this amino acid were extracted following 1-h extraction with deionised water and citric acid in the case of *A. nodosum* and *F. vesiculosus*, respectively.

Studies have shown that the amounts of FAAs vary amongst seaweed species. Estimation of FAAs from *C. crispus*, *Porphyra *spp., *P. palmata*, *A. nodosum*, *F. vesiculosus*, *H. elongata*, *Laminaria *sp., *U. pinnatifida* and *Ulva* sp. was carried out, and it was observed that the brown seaweeds had higher FAAs followed by green and red seaweeds (Vieira et al. 2018)*.* FAAs from *Porphyra dioica*, *Porphyra umbilicalis*, *Gracilaria vermiculophylla* and *Ulva rigida* were obtained by aqueous extraction involving agitation at room temperature for 30 min (Machado et al. 2020). In a study, FAAs were extracted from alga *Pyropia yezoensis* using ethanol (96% ethanol (1:20 w/v), 25 °C), hot water extraction (100 °C, 3 h) and subcritical water extraction (120–230 °C, 30 bar) (Park et al. 2019). It was reported that both hot water extraction and subcritical water extraction at 120 °C were suitable for extraction of FAAs and that temperature had a significant impact on the total amino acids as well as FAAs obtained.

### Umami FAAs

Extraction of umami FAAs from various sources utilising different methods including water-based extraction, fermentation, ultrasound and acid hydrolysis was outlined by Zhao et al. (2019). However, limited information is available in the scientific literature on the levels of umami FAAs in seaweed (Mouritsen et al. 2012, 2019; Hamid et al. 2018; Stévant et al. 2018; Poojary et al. 2019; Milinovic et al. 2020).

**Fig. 2:**
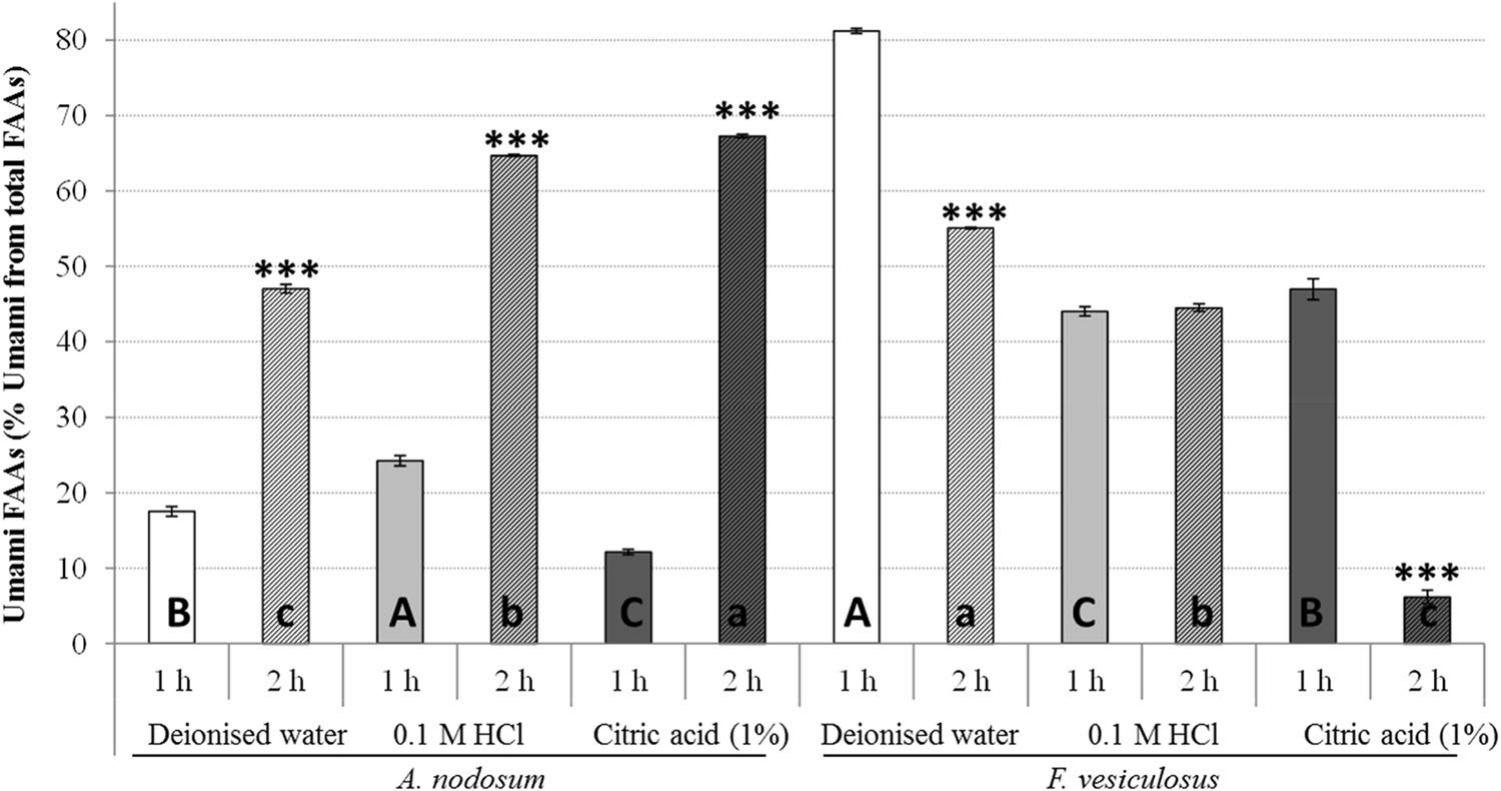
Bar charts representing the influence of solvent type (deionised water, 0.1 M HCl and 1% citric acid) and extraction time (1 and 2 h) on the % umami FAAs extracted from *A. nodosum* and *F. vesiculosus.* Results are expressed as average ± standard deviation of the mean. Different letters indicate statistical differences in the % of umami FAAs extracted using multiple solvents during 1 h (uppercase letters) or 2 h (lowercase letters). The statistical differences in the % of umami amino acids extracted by using different times of extraction and the same solvent are represented as: *P < 0.05, **P < 0.01 and ***P < 0.001

The amount of umami FAAs extracted from *A. nodosum* and *F. vesiculosus* for each extraction condition used in the current study, expressed as a % of umami FAAs from the total FAAs, is shown in Fig. 2. In general, the extracts obtained from *A. nodosum* had lower amounts of umami FAAs compared to those extracted from *F. vesiculosus*, with variable effects of extraction solvent and time observed*.* In the case of *A. nodosum*, increased extraction time resulted in increased yield of umami FAAs independently of solvent type used. However, in the case of *F. vesiculosus*, an increased extraction time had a negative effect on the yield of umami FAAs when using water and citric acid solvents and no effect when using 0.1 M HCl solvent. When evaluating the efficiency of the solvents, the highest recoveries of umami FAAs were observed when using citric acid and deionised water in *A. nodosum* and *F. vesiculosus*, respectively. Thus, both solvents are suitable for use as clean extraction solvents to replace HCl to extract flavoured compounds from seaweeds.

In recent studies, 0.1 M HCl is one of the most commonly used solvents to extract umami compounds from plant sources such as mushrooms (Phat et al. 2016; Poojary et al. 2017). Similarly to the results of this study, the extraction of umami FAAs from the microalga *Chlorella vulgaris* was improved by using distilled water as extraction solvent compared to 0.4 M HCl using ultrasonic equipment (Hildebrand et al. 2020). Moreover, when extracting umami FAAs from other matrices, such as mushrooms, using water as solvent also achieved higher yields of umami FAAs compared to 0.1 M HCl (Poojary et al. 2017). In general, umami FAAs and the umami peptides are water soluble, and thus, the use of water as solvent which has significant economic and environmental benefits over other solvents will be favoured when extracting these compounds at large scale.

**Fig. 3:**
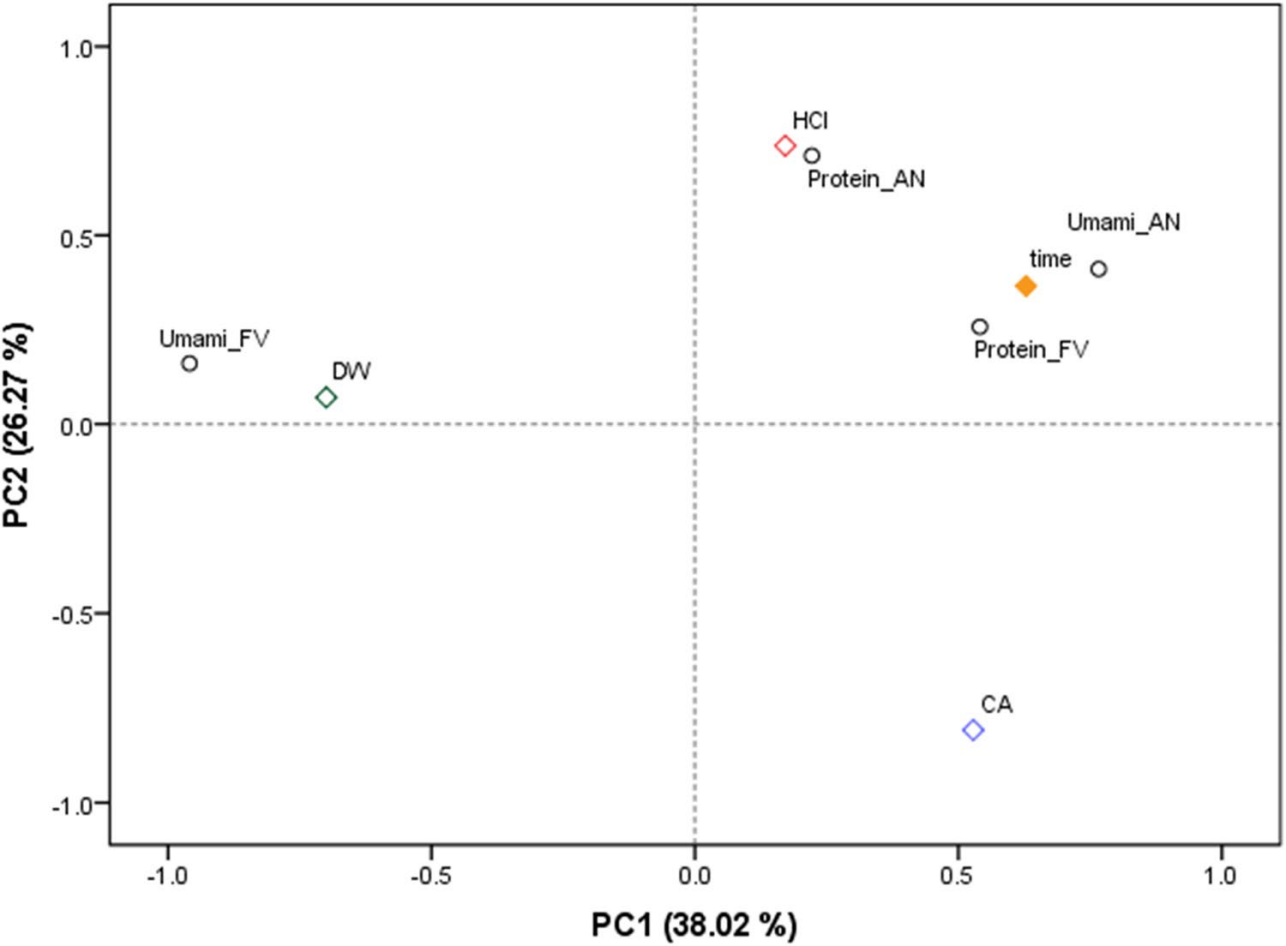
PCA scatter plot representing the scores for the recovery of umami FAAs and protein from *A. nodosum* (AN) and *F. vesiculosus* (FV). The solvents of extraction were abbreviated in the figure as follows: DW (deionised water), HCl (0.1 M HCl) and CA (1% citric acid)

PCA was performed to analyse the similarities and differences in the recovery of umami FAAs and protein from the two brown macroalgal species depending on the different extraction conditions applied. The principal components PC1 and PC2 accounted for an overall 64.29% of the total variance in the data set (Fig. 3). PC1 (38.02%) seems to separate the acidic solvents (HCl and citric acid) from that of deionised water that appeared clustered to the recovery of umami FAAs from *F. vesiculosus* in the left side of PC1. PC2 (26.27%) separates further the data set and clustered the recovery of umami FAAs from *A. nodosum* and protein from *F. vesiculosus* with the time of extraction, while the recovery of protein from *A. nodosum* is clustered with the use of 0.1 M HCl. These results confirm the different behaviour in the recovery of compounds between both seaweed species that could be attributed to interspecific differences in the polysaccharides produced by both species (Harnedy and FitzGerald 2013) and thus, different ionic interactions influencing the yields and behaviour of extraction of the compounds of this study.

## Conclusions

The brown macroalgae investigated in this study can be considered feasible sources of protein and FAAs including umami FAAs. The extraction of protein using different solvents and extraction time was not significantly influenced by the process parameters studied; however, these process parameters strongly influenced the amount of FAAs and umami FAAs extracted from *A. nodosum* and *F. vesiculosus*. The extraction yield for all compounds was significantly influenced by the seaweed species investigated, highlighting that inter-species differences in protein and other cell wall compounds interacting with the targeted compounds of extraction need to be considered when designing industrial extraction protocols. For the extraction of proteins and FAAs, the maximum recovery of these compounds was achieved by extracting from the biomass for 2 h with deionised water or 1% citric acid for *A. nodosum* and *F. vesiculosus*, respectively. In the case of umami FAAs from *A. nodosum*, optimum yields were achieved by extracting the biomass using 1% citric acid for 2 h, while in the case of *F. vesiculosus*, the use of water solvent and 1 h of extraction time resulted in the optimum yield. Further studies investigating the effect of additional process parameters (i.e. solid:liquid ratio and temperature), as well as other seaweed species and agri-food by-products or bioresources as sources of flavoured agents, are recommended. Overall, this study demonstrates the potential of using green solvents (deionised water and citric acid) for the recovery of proteins, FAAs and umami FAAs, and will facilitate the adoption of more sustainable approaches in industrial-scale extraction operations for the recovery of flavoured compounds from seaweeds.

## Data Availability

All data generated or analysed during this study are included in this published article.
